# Effect of Osmotic Stress on the Growth, Development and Pathogenicity of *Setosphaeria turcica*

**DOI:** 10.3389/fmicb.2021.706349

**Published:** 2021-07-23

**Authors:** Yuwei Liu, Xiaodong Gong, Moxiao Li, Helong Si, Qihui Zhou, Xingchen Liu, Yu Fan, Xiaoyu Zhang, Jianmin Han, Shouqin Gu, Jingao Dong

**Affiliations:** ^1^State Key Laboratory of North China Crop Improvement and Regulation, Baoding, China; ^2^College of Life Sciences, Hebei Agricultural University, Baoding, China; ^3^Key Laboratory of Hebei Province for Plant Physiology and Molecular Pathology, Baoding, China; ^4^College of Plant Protection, Hebei Agricultural University, Baoding, China

**Keywords:** *Setosphaeria turcica*, osmotic, pathogenicity, HOG-MAPK, *StFPS1*

## Abstract

Osmotic stress is a severe condition frequently encountered by microorganisms; however, there is limited knowledge on the influence of hyperosmotic stress on the growth, development and pathogenicity of phytopathogenic fungi. Here, three osmotic conditions (0.4 M NaCl, 0.4 M KCl, and 0.6 M sorbitol supplemented in potato dextrose agar medium) were used to identify the effect of osmotic stress on the growth, development and pathogenicity of *Setosphaeria turcica* which is a plant pathogenic fungus and causes northern corn leaf blight disease in maize, sorghum, and related grasses. In osmotic stress, the growth rate of mycelium was decreased, and the number of vesicular structures and flocculent secretion outside the hypha cell wall were significantly increased. The qRT-PCR results showed that the osmotic stress quickly activated the HOG-MAPK pathway, up-regulated the expression of the downstream genes, and these genes were most highly expressed within 30 min of exposure to osmotic stress. Furthermore, the germination rate and the yield of conidia were significantly higher under osmotic stress than in the control. A pathogenicity analysis confirmed that pathogenicity of the conidia which were cultured under osmotic stress was significantly enhanced. By analyzing the knock-out mutants of an osmotic stress responsed gene *StFPS1*, an aquaglyceroporin downstream of the HOG-MAPK pathway, we found that *StFPS1* was involved in the formation of appressorium and penetration peg, which affected the penetration ability of *S. turcica*. In summary, our work explained the correlation between osmotic stress and growth, development, and pathogenicity in *S. turcica*.

## Introduction

Organisms are constantly exposed to abiotic stress factors, such as ultraviolet radiation, temperature fluctuations, osmotic imbalance and oxidative damage, during their life cycle (Gasch et al., 2000; Duran et al., 2010; Abu Bakar et al., 2020). Hyperosmotic stress is a prevalent, adverse condition that could cause osmotic dehydration and cell death (Bremer and Krämer, 2019).

Previous studies have shown that exposure of microorganisms to specific incremental stress enhances their resistance to that particular stress factor and other stresses. This is known as cross-protection or cross-resistance (Gasch, 2003). Therefore, evaluating the cellular response induced by stress could reveal the mechanisms by which the cell responds to other stress factors (Brown et al., 2014). You et al. (2012) showed that cells can “remember” previous hyperosmotic stress conditions for some time beyond the duration of signal transduction, and thus they can quickly adapt to subsequent exposure. Filamentous fungi exposed to priming stress show a better response to temperature stress than those that have not been exposed to priming stress (Andrade-Linares et al., 2016).

When exposed to stress, the fungi initiate a series of reprogramming processes to adapt to the environmental changes. For example, under hyperosmotic stress conditions, the hyphae of *Glomus intraradices* grow in a curled hyphae instead of the typical straight runner hyphae and the spore number was significantly decreased (Hammer and Rillig, 2011). In *Ashbya gossypii*, it was found that the growth rate and the total biomass were significantly decreased in the medium supplemented with NaCl (Förster et al., 1998). At the cellular level, hyperosmotic stress causes water outflow from the cell, an increase in cytoplasmic ion concentration, and the contraction of protoplasts (Saito and Posas, 2012). In addition, the molecular regulation of cellular responses to osmotic pressure has been well studied. The high osmolarity glycerol mitogen-activated protein kinase (HOG-MAPK) pathway, which is composed of Ste11/Ssk2/Ssk22 (MAPKKK) -Pbs2 (MAPKK)-Hog1 (MAPK), was reported to be able to govern adaptive responses in fungi (Hohmann, 2002). Among of these components, Hog1 is the central core of the HOG-MAPK cascade (Saito and Posas, 2012). Deletion of Hog1-encoding genes in *Bipolaris oryzae* (Moriwaki et al., 2006), *Magnaporthe oryzae* (Dixon et al., 1999), *Ustilaginoidea virens* (Zheng et al., 2016), and *Fusarium graminearum* (Zheng et al., 2012) cause a defect in adaptation to high osmolarity conditions. Furthermore, it was reported that the homologs of Hog1 were involved in species and tissue-specific pathogenesis (Jiang et al., 2018). For example, Osm1 which is the homolog of Hog1 in *M. oryzae* is dispensable for pathogenesis (Dixon et al., 1999), while, our previous research demonstrated that a MAPK gene named *StSTK1* is essential for hyphal, conidial, and appressorial development; toxin biosynthesis; pathogenicity; and osmotic stress reaction in the plant pathogenic fungus *Setosphaeria turcica* (Li et al., 2016).

Although the osmotic stress response and cellular memory phenomena have been studied in fungi (You et al., 2012), the research of the correlation between osmotic stress and pathogenicity is rarely reported in plant pathogenic fungi. In the present study, the effect of osmotic stress on the growth and development, and pathogenicity in *S. turcica* was explored. Further, the functions of the osmotic stress-related gene *StFPS1*, an aquaglyceroporin gene downstream of the HOG-MAPK pathway, was investigated to confirm the relationship between osmotic stress and pathogenicity. This research will provide an example for understanding the correlation between osmotic stress and pathogenicity in plant pathogenic fungi.

## Materials and Methods

### Strains and Growth Conditions

The strain of *S. turcica* used in the present study was 01–23. The strain was cultured according to our previous study with minor modifications (Gu et al., 2014). Specifically, potato dextrose agar (PDA) medium supplemented with 0.4 M NaCl, 0.4 M KCl, or 0.6 M sorbitol was used for the osmotic stress treatments (Araújo et al., 2020). Mycelium of *S. turcica* with a diameter of 0.8 cm was inoculated with the above four media, respectively, (each medium had three replicates) in darkness at 25°C and the colony diameters were measured from the first day to the sixth day after inoculation. All tests were performed in three independent times.

### Colony Growth Rate, Yield of Conidium, Hyphae and Conidia Morphology

Colony growth rate was monitored and calculated using the method reported in our previous research (Gu et al., 2014). After 20 days of culture under darkness at 25°C, the effects of osmotic stress treatment on the morphology of aerial hyphae, basal hyphae, and conidium were observed. Aerial hyphae from the four media types (each medium had three replicates) were selected from the colony edges and observed under a microscope (Nikon eclipse, E-200). After scraping off the aerial hyphae on the media surfaces, the morphology of the basal hyphae and conidium were examined at 10× and 40× magnifications. Conidium yield was evaluated using a hemocytometer according to our previous report (Zhang et al., 2003), and performed in three independent times.

### Appressorium Formation and Penetration Assay

The strain was cultured on PDA medium at 25°C in the dark for 20 days. 5 mL of H_2_O, 1.1 M NaCl, 1.1 M KCl, and 1.3 M Sorbitol were added on the surface of the colonies to obtain conidium suspensions and each of the above solution was performed in triplicate. These suspensions were incubated at 25°C for 30 min and 3 h and then the conidia concentration was adjusted to 10^4^/mL using ddH_2_O. The suspensions were then placed on water agar media covered with cellophane and incubated in the dark at 25°C. The germination of conidia and the formation of invasive structures were observed 3, 6, 9, 11, 12, 24, 36, 48, 60, and 72 h post cultivation. At least 10 sights of each sample were observed. Artificial cellophane film was used for the penetration assay, as detailed in our previous report (Gong et al., 2021).

### Evaluation of the Pathogenicity on Maize Seedlings

The strain was cultured on PDA medium and PDA medium supplemented with 0.4 M KCl at 25°C in the dark for 20 days. Then, 5 mL H_2_O was added on the surface of the colony to make a conidial suspension and the conidial concentration was adjusted to 10^4^/mL. Further, 200 μL of suspension was inoculated on three maize seedling whorls at the 4–6 leaf stage. Inoculated seedlings were placed in a dark growth chamber with 80% humidity for 24 h and cultivated in 12 h of light, 12 h of darkness at 25°C for 7–15 days to evaluate the pathogenicity on maize leaves.

### Observation of Cell Wall Ultrastructure

After 3 days of growth in PDA medium or PDA medium supplemented with 0.4 M NaCl, the aerial and basal hyphae were sampled and quickly placed in a 1.5 mL EP tube containing the initial fixative solution [2.5% glutaraldehyde (w/v) made up in 0.1 M phosphate buffer (pH 7.2)]. The methods for scanning electron microscopy and transmission electron microscopy were those presented in our previous report (Gong et al., 2017a). At least 10 cell were observed for each sample.

### Expression Analysis of Key Genes in the *S. turcica* HOG-MAPK Pathway Under Osmotic Stress

The search of HOG-MAPK pathway homologous genes in *S. turcica* was using the local Blastp program (version 2.7.1+). The protein sequences related to HOG-MAPK pathway in *S. cerevisiae* were as queries to search the GeneCatalog proteins^1^ of *S. turcica*. For the blast results, the best alignment of *S. turcica* protein was regarded as the homolog of *S. cerevisiae*. For the qRT-PCR analysis, the strain was sampled during active growth. A mycelium with a diameter of 0.8 cm was inoculated in potato dextrose (PD) medium and incubated in the dark at 25°C for 5–7 days. The PD medium was poured out and completely drained off between filter papers. Then, the pathogen was cultivated in PD medium supplemented with 0.4 M NaCl for 0, 5, 10, 15, 20, and 30 min, and 1, 3, 12, 24, 36, 48, and 72 h. Subsequently, the pathogen was taken out and the excess water was removed using filter paper. RNA extraction was conducted using the UNIQ-10 column Trizol total RNA extraction kit following the manufacturer’s protocol (Songon Biotech, China). Total RNA was reverse transcribed to complementary DNA (cDNA) using M-MLV reverse transcriptase (Promega) according to the manufacturer’s instructions. The qRT-PCR analysis was performed using TransStart Top Green qPCR SuperMix (TransStart, China) and the primers are shown in Supplementary Table 1. PCR reactions were performed in triplicate for each sample.

### Transformation-Mediated Gene Disruption

Gene knock-out was done based on a previously published method (Ruiz-Roldán et al., 2001). Briefly, an upstream flanking fragment of *StFPS1* (*StFPS1-A*) was amplified by StFPS1-AL/StFPS1-AR primers, while StFPS1-BL/StFPS1-BR was used to amplify a downstream flanking fragment of *StFPS1* (*StFPS1-B*). After amplicon purification using the TaKaRa DNA Fragment Purification Kit ver. 2.0 (TaKaRa, Japan), the fragments *StFPS1-A* and *StFPS1-B* were cloned into the vector pBS, which confers resistance to hygromycin B phosphotransferase. The resulting plasmid, named pBS-A-HPH-B, was used to transform wild-type (WT) of *S. turcica* protoplasts. The transformed protoplasts were incubated on PDA medium containing hygromycin B (50 μg/mL) at 25°C for 6–7 day until hygromycin B-resistant transformants were observed. All transformants were individually preserved and subjected to verification by PCR using the primers *HPH*-L/*HPH-*R and a primer which was in the upstream of *StFPS1* (*StFPS1*-UpL) and a primer in *HPH* (*HPH*-UpR).

### HT-Toxin Isolation and HPLC Analysis Assay

After 21 day of culture in the dark at 25°C, the mycelia of Δ*StFPS1* and WT were filtered away with 4-layer gauze. The filtrate was centrifuged (2,000 × *g*, 10 min), and the supernatant was extracted with acetic ether ethyl acetate (AEEC) at 25°C and 200 r min^–1^ for 12 h. The water-soluble extract was further extracted with AEEC. The residue was dissolved in methanol and used in the subsequent HPLC analysis and toxicity assay, as described previously (Zhang et al., 2007).

## Results

### Osmotic Stress Affects the Growth of *S. turcica*

**FIGURE 1:**
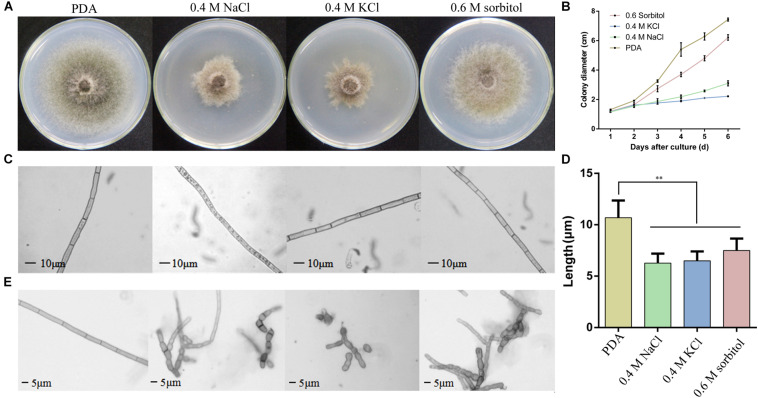
Observation of the *Setosphaeria turcica* phenotype when cultured under osmotic conditions. **(A)** Colony morphology and **(B)** the growth rate of *S. turcica* in potato dextrose agar (PDA) and 0.4 M NaCl, 0.4 M KCl, and 0.6 M sorbitol culture media. The results are shown as mean colony diameter from three independent experiments. **(C)** Morphology and **(D)** the growth rate of aerial hyphae under the above four culture mediums; **(E)** morphology of basal hyphae under the above four culture conditions. These results are shown as mean length of hyphae from three independent experiments. Data are presented as the mean ± SD of three independent experiments. **P < 0.01.

To determine the effects of osmotic conditions on the growth and development of *S. turcica*, the strain was inoculated into PDA medium supplemented with 0.4 M NaCl, 0.4 M KCl, and 0.6 M sorbitol. Firstly, the colony diameter was measured every day after inoculation and the colony morphology was observed on the sixth day. The results showed poor hypha growth under osmotic stress (Figure 1A). Compared with the 0.6 M sorbitol treatment, the colonies were significantly limited under the 0.4 M NaCl and 0.4 M KCl treatments (Figure 1B), indicating that the hypha are more sensitive to ionic stress than non-ionic stress. Furthermore, obvious granular substances were observed in the hyphae of *S. turcica* in the 0.4 M NaCl treatment (Figure 1C). In addition, it was found that osmotic stress had a significant effect on the growth of the basal hyphae. The hypha septum of *S. turcica* in the medium supplemented with 0.4 M NaCl, 0.4 M KCl, and 0.6 M sorbitol were significantly shorter than control (Figure 1D). Some of the hyphae even expanded to form a spherical shape (Figure 1E). Overall, these results indicate that both aerial and basal hyphae exhibited short turns and swellings to various degrees when cultivated in osmotic media.

**FIGURE 2:**
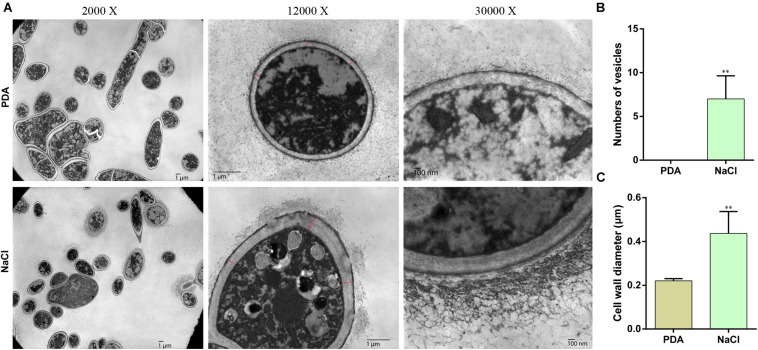
Transmission electron microscopy (TEM) of hyphae of *S. turcica* cultured on PDA and 0.4 M NaCl. **(A)** Representative TEM images (magnification, ×2,000, ×12,000, and ×30,000, respectively) of hyphae. **(B)** The mean number of vesicles in each cell. **(C)** The mean diameter of the cell wall. Data are presented as the mean ± SD of each cell. **P < 0.01.

Prompted by the granular substances observed in hyphae grown on PDA medium with 0.4 M NaCl, we determined whether osmotic stress affects the formation and transport of vesicles. Therefore, following 3-day culture in PDA and PDA supplemented with 0.4 M NaCl, hyphae were observed via electron microscopy (Figure 2A). The hyphae cultivated under osmotic conditions produced numerous membrane vesicles, whereas this was not found in cells cultured in PDA alone (Figure 2B). In addition, the hypha cell wall in the osmotic medium became significantly thicker and the flocculent secretions outside the cell wall increased compared with those in the control (Figure 2C). These results indicated that vesicle transport was increased under osmotic stress in *S. turcica*.

### Identification of Genes Responsive to Osmotic Stress in *S. turcica*

**FIGURE 3:**
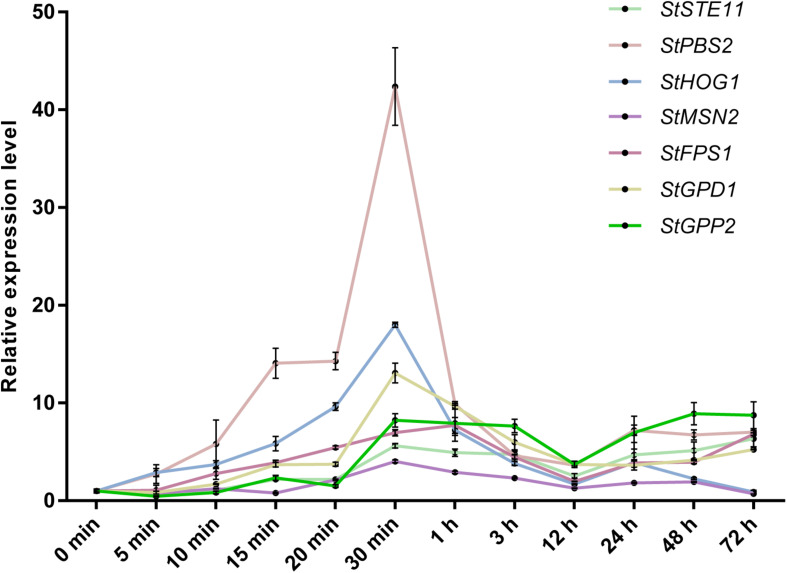
qRT-PCR analysis of the relative expression of HOG-MAPK related genes at different treatment times in 0.4 M NaCl treatment. Data are presented as the mean ± SD of three independent experiments.

In light of the osmotic stress has a great influence on the growth of *S. turcica*, we wanted to explore the genes that can respond to hypertonic stress. Previous studies have indicated that HOG-MAPK pathway contributes to osmotic regulation (Hohmann, 2002). To explore whether the HOG-MAPK pathway can respond to osmotic stress in *S. turcica*, the dynamic changes of gene expression related to HOG-MAPK pathways were studied. Specifically, seven genes involved in the HOG-MAPK pathways, namely *StSTE11* (Locus ID from Joint Genome Institute: 158652), *StPBS2* (138151), and *StHOG1* (47672), and the downstream genes of the HOG-MAPK cascade, namely *StMSN2* (101099), *StFPS1* (153214), *StGPD1* (166570), and *StGPP2* (1414693), were evaluated. The expression levels of *StPBS2*, *StHOG1*, *StMSN2*, *StFPS1*, and *StGPD1* increased first and then decreased with the increase in osmotic stress treatment time (0.4 M NaCl; Figure 3). The mRNA level of *StFPS1* was highest after 1 h of osmotic treatment, whereas the mRNA levels of the other four genes reached a peak value after 30 min of osmotic treatment. Notably, the relative expression level of *StHOG1* and *StMSN2* decreased to its lowest level after 72 h of osmotic stress treatment (Figure 3). This finding agrees with the previous report that to adapt to osmotic stress, yeast cells re-establish osmotic balance and decrease the relative expression level of *StHOG1* to its lowest level (Saito and Posas, 2012). In addition, the results showed that the expression of almost all the tested genes was significantly reduced at 12 h after osmotic stress treatment (Figure 3). These results indicate that both the HOG-MAPK pathway genes and the key downstream genes respond to osmotic stress via dynamic gene transcription in *S. turcica*.

### Osmotic Stress Strengthens the Formation of Infection Structures

**FIGURE 4:**
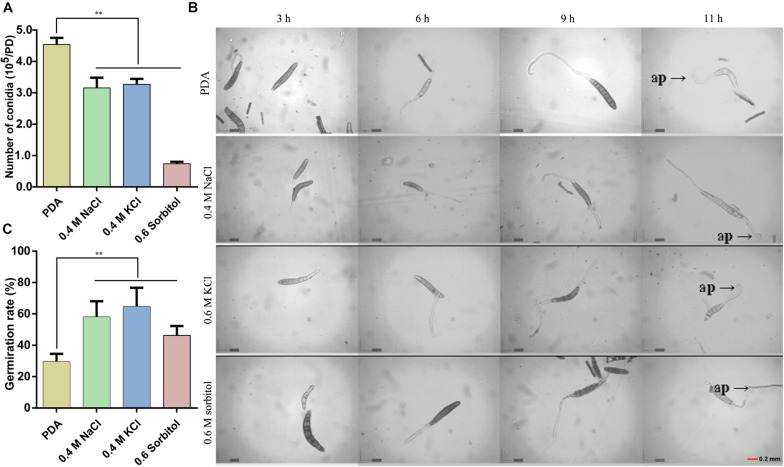
Observation of conidia germination after 20 days of culture under the three osmotic conditions (0.4 M NaCl, 0.4 M KCl, and 0.6 M sorbitol) and control (PDA). **(A)** The number of conidia produced by the hyphae under the four media. **(B)** The conidial suspension was incubated on cellophane at 25°C in the dark and observed at 3, 6, 9, and 11 h after inoculation under a microscope and **(C)** the quantitative statistics from three independent petri dishes. ap: appressorium, Data are presented as the mean ± SD of three independent experiments.**P < 0.01.

**FIGURE 5:**
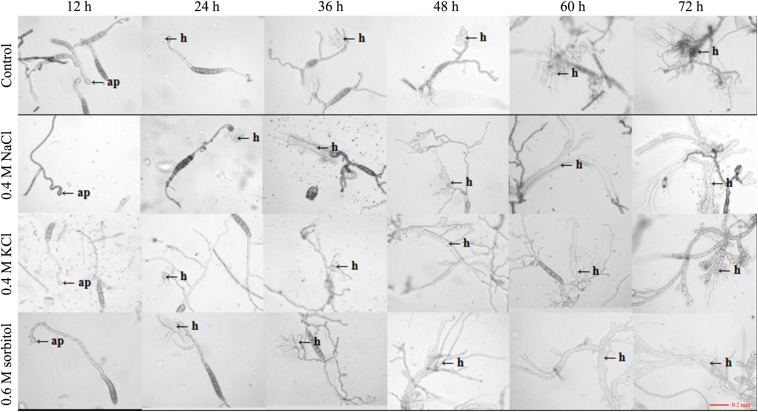
Observation of appressorium development after 20 days of culture under the three osmotic conditions (0.4 M NaCl, 0.4 M KCl, and 0.6 M sorbitol) and control (PDA). The conidial suspension was incubated on cellophane at 25°C in the dark and observed at 12, 24, 36, 48, 60, and 72 h after inoculation under a microscope. h: invading hyphae.

The HOG pathway plays a species-specific role in pathogenesis (Jiang et al., 2018). In our previous study, we found the *StHOG1* was required for pathogenicity in *S. turcica* (Li et al., 2016). Therefore, the strong transcriptional induction of *StHOG1* in osmotic stress led us to consider the possibility that osmotic stress have a certain relationship to pathogenicity. To test this, the germination of conidia and appressorium formation and the formation of infection structures which cultured on PDA medium with different osmotic stress conditions were observed. We found that the hyphae could induce conidia under different culture conditions, but the conidia yield was significantly decreased (*P* < 0.05) under hypertonic culture (Figure 4A). Among of them, the conidia yield was the lowest in the medium supplemented with 0.6 m sorbitol (Figure 4A). In addition, the conidium could germinate after 3 h of cultivation in the PDA medium and the three osmotic media (Figure 4B). In the control group (PDA alone), the germination rate of conidia was 29.51%, whereas the germination rate in the media supplemented with 0.4 M NaCl, 0.4 M KCl, and 0.6 M sorbitol were 58.08, 64.63, and 46.30%, respectively, which were significantly higher than the PDA control (*p* < 0.01; Figure 4C). However, osmotic stress did not affect the conidial morphology and similar to the control, most conidium was fusiform with six to eight compartments (Supplementary Figure 1). Further, whether osmotic conditions affect the formation of infective structures was also studied. The formation of penetration pegs could be found both in the control and the three osmotic treatments after 12 h of induction (Figure 5). However, the morphology of invading hyphae differed between the control and osmotic conditions. Under osmotic conditions, the invading hyphae generally gathered into a bunch, grew rapidly toward a specific direction, and the tip continued to bifurcate and extend forward at 60 and 72 h. Meanwhile, the invading hyphae formed in PDA grew radially around the penetration peg (Figure 5). In addition, the higher osmolality (1.3 M NaCl) with shorter-term (3 h) osmotic stress treatment had the same effect on the morphology of invading hyphae in the 20-day 0.4 M NaCl treatment. However, there was no obvious difference among the morphology of hyphae in the other treatments (Supplementary Figure 2). These results suggest that osmotic stress influences the germination of conidia and the morphology of invading hyphae.

### Osmotic Stress Enhances the Pathogenicity of *S. turcica*

**FIGURE 6:**
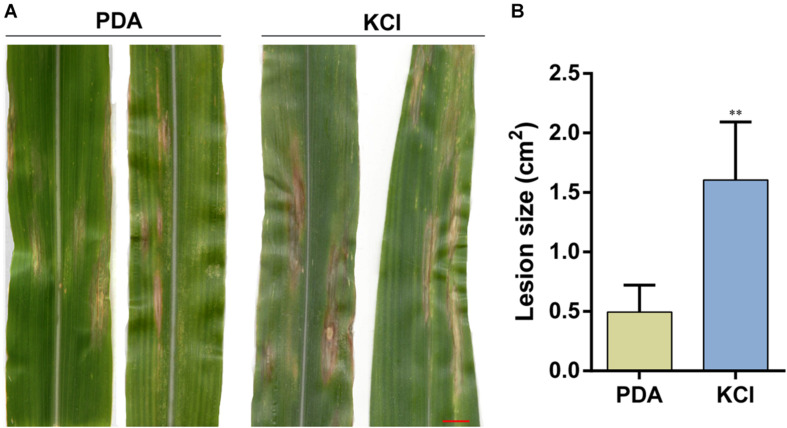
Pathogenicity of *S. turcica* after 20 days of culture under the 0.4 M KCl and control (PDA). **(A)** Conidial suspensions were inoculated on maize B73 seedling whorl at the 4–6 leaf stage, and results were scored at 12 days post-inoculation and **(B)** the mean size of the lesion. **P < 0.01, bar = 1 cm.

To confirm the effect of osmotic stress on the pathogenicity of *S. turcica*, host infection experiments were performed. Leaf whorls of maize seedlings at the 4–6 leaf stage were inoculated with conidial suspension supplemented with 0.4 KCL or not. The results indicated that plants inoculated with conidial suspension supplemented with KCl showed 1–2 days earlier disease spots. Furthermore, the disease spots on these plants were expanded than on plants inoculated with conidial suspension not supplemented with KCl 12 days post inoculation (Figure 6). These results suggest that osmotic stress enhanced the pathogenicity of *S. turcica* in maize.

### Osmotic Stress Related Gene *StFPS1* Regulates the Invasiveness of *S. turcica*

**FIGURE 7:**
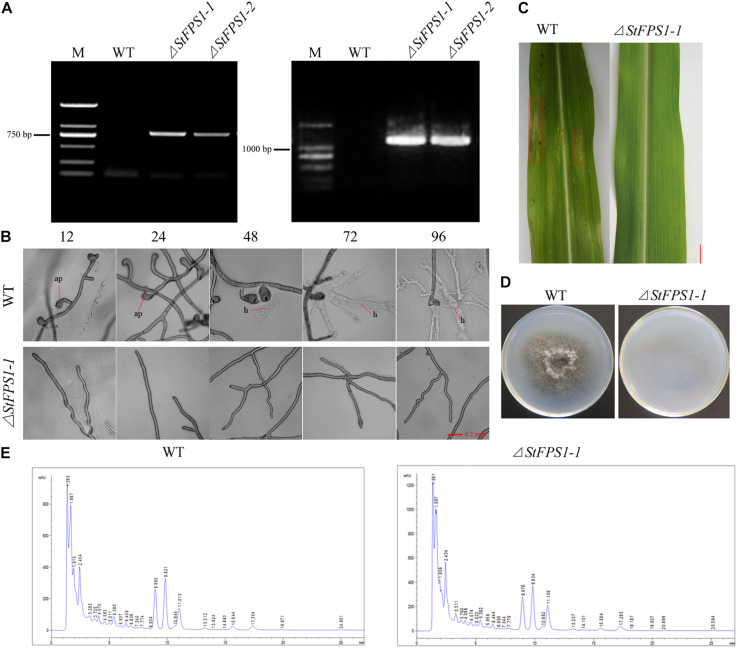
Identification of the *StFPS1* knockout mutant (Δ*StFPS1*) and functional analysis of Δ*StFPS1*. **(A)** *S. turcica* wild-type (WT) and Δ*StFPS1*, identified by amplifying a fragment of the hygromycin B phosphotransferase gene (*HPH*) using specific primers (*HPH*-L/*HPH-*R; left) and fragment including the *StFPS1* upstream flanking fragment and *HPH* using specific primers (*StFPS1*-UpL/*HPH*-UpR; right). **(B)** Observation of the appressorium development of WT and Δ*StFPS1* at 12, 24, 48, 72, and 96 h after inoculation under a microscope. **(C)** Mycelial suspensions were inoculated on maize B73 seedling whorl at the 4–6 leaf stage, and results were scored at 10 days post-inoculation, bar = 1 cm. **(D)** Comparison of the cellophane penetrating ability of WT and Δ*StFPS1* after the strains were incubated on cellophane at 25°C in the dark for 5 days and then removed the cellophane and continued to incubate for 2 days for observation. **(E)** High performance liquid chromatography analysis of HT-toxins of WT and Δ*StFPS1*.

Previous study of Luyten et al. (1995) showed that Fps1, an aquaglyceroporin of yeast, facilitates glycerol transport in response to changes in extracellular osmolarity. Our results indicated that *StFPS1*, a homologous of yeast *FPS1*, was up-regulated during osmotic stress (Figure 3). To investigate the effect of osmotic related gene *StFPS1* in pathogen pathogenicity, mutant strain of *S. turcica*, namely, Δ*StFPS1* was generated via protoplast transformation and confirmed by PCR analysis (Figure 7A). The results showed that the hyphae of Δ*StFPS1* could not form any appressorium and penetration pegs after 84 h of induction, while, the appressorium and penetration pegs of the WT strain were observed after 24 h and 36 h of induction (Figure 7B). These results suggest that *StFPS1* significantly influences the formation of infective structures. To evaluate whether *StFPS1* is essential for pathogenicity, the mycelial suspensions of WT and Δ*StFPS1* were inoculated on the maize leaves. 10 days after inoculation, the maize leaves exhibited typical fusiform lesions after inoculation with WT, while no lesions were detected after inoculation with Δ*StFPS1* (Figure 7C), indicating *StFPS1* is required for pathogenesis of *S. turcica*. In order to clarify the influence of *StFPS1* on pathogenicity, a penetration test was then performed to confirm the influence of *StFPS1* on penetrability. Hyphae taken from the WT strain and Δ*StFPS1* were separately inoculated on PDA media covered with artificial cellophane film which can simulate hyphal penetration into maize leaves. The results showed that Δ*StFPS1* could not penetrate the cellophane to form a colony, while the WT strain showed the opposite results (Figure 7D). To further elucidate whether *StFPS1* influence the pathogenicity of *S. turcica*, the components of crude HT-toxin which is an important pathogenic factor in *S. turcica* were analyzed by HPLC (Gu et al., 2014). The results showed that the Δ*StFPS1* and WT strains had absorption peaks at the same time, and the peak areas were almost the same, indicating that the deletion of *StFPS1* did not change the composition and content of HT-toxin (Figure 7E). Based on the above results, we inferred that *StFPS1* effects the development of appressorium and the penetration ability of *S. turcica*.

## Discussion

To adapt to hyperosmotic stress, microorganisms have formed a set of effective osmoadaptation mechanisms over the course of evolution. One of the most common mechanisms of osmoadaptation mechanisms is the production and accumulation of osmoregulatory substances to maintain intracellular water balance (Saito and Posas, 2012). In *S. turcica*, our previous study confirmed that hyperosmotic stress significantly changed the contents of glycerol, trehalose and mannitol in mycelial cells (Gong et al., 2017b). To further explore the effect of osmotic stress on the growth, development, and pathogenicity in *S. turcica*, the pathogen was exposed to NaCl, KCl, and sorbitol. Relative to sorbitol, both NaCl and KCl severely inhibited pathogen growth (Figure 1A). These findings are in accordance with the study on hyperosmotic stress in *Ashbya gossypii*, which reported that the growth rate of NaCl-treated cells was significantly decreased, whereas there was a minor effect on grown in the mannitol treatment (Förster et al., 1998). Further analysis found that many granular substances were in the hyphae which were cultured on medium with 0.4 M NaCl, while it was not found in other cases. These results further confirm that osmotic stress caused by Na^+^ could have an additional influence on the content in the hyphae (Figure 1B). This finding is similar to a previous report revealing that sodium toxicity poses additional influence on the growth of *S. cerevisiae* (Prista et al., 2005). Furthermore, electron microscopy demonstrated a significant influence of osmotic conditions on the morphology of *S. turcica* cells. Therefore, cell resistance to external osmotic stress conditions involves multiple coordinated responses.

The osmoadaptation mechanism in yeast has been studied intensively, and the HOG-MAPK pathway is the main pathway that acts in response to hyperosmotic stress. Moreover, the role of HOG-MAPK in osmoregulation is conserved in many fungi, such as in *Verticillium dahliae* (Wang et al., 2016), *B. oryzae* (Turrà et al., 2014), *U. virens* (Zheng et al., 2016), and *S. turcica* (Li et al., 2016). In yeast, HOG-MAPK mediated osmoregulation by phosphorylating the activation site of Hog1 (peaking at 5 min), after which most Hog1 are transported to the nucleus to regulate transcription and the cell cycle, and after 30 min, glycerin accumulates in the cell to produce high osmotic pressure (Hao et al., 2007; Muzzey et al., 2009; Wosika and Pelet, 2020). In addition, in yeast, the greatest changes in the high osmolarity-regulated genes were observed within the first 40 min of treatment, with the maximal induction after 20 min of treatment (O’Rourke and Herskowitz, 2004). In the present study, genes related to the HOG-MAPK pathway participate in osmotic stress response via transcriptional regulation (Figure 2). The results of this study showed that most of these genes were maximally up-regulated within 30 min of osmotic treatment, suggesting that the HOG-MAPK pathway-related genes could rapidly respond to osmotic stress at the mRNA level (Figure 2). In addition, some reports have focused on the influence of osmotic stress on secondary metabolism and/or morphogenesis, such as in the yeast *S. cerevisiae*, *Aspergillus species* (Hohmann et al., 2007; Miskei et al., 2009). However, few reports focus on the influence of osmotic stress on the pathogenicity in plant pathogenic fungi. It’s worth noting that the HOG-MAPK pathway has species-specific differences in pathogenesis of plant pathogenic fungi (Dixon et al., 1999; Wang et al., 2016; Segorbe et al., 2017; Jiang et al., 2018). In *S. turcica*, for instance, we previously found that the MAPK gene *StHOG1* is required for pathogenicity (Li et al., 2016). In the present study, we further found that the downstream gene *StFPS1* is also involved in regulating fungal invasiveness (Figure 7). A previous study on yeast showed that the Fps1p channel plays an important role in facilitating glycerol efflux (Beese-Sims et al., 2011). Therefore, it is possible that *StFPS1* reduces glycerol transport and the hyphae subsequently lack the pressure to form infective structure. For *M. oryzae*, the appressorium develops a high internal turgor pressure for penetration of the rice leaf and glycerol accumulation plays an important role in appressorium turgor (Foster et al., 2017). These results demonstrate the significance of the HOG-MAPK pathway in fungal development and pathogenicity.

Cross-protection is a common phenomenon in microorganisms. For instance, in *Zygosaccharomyces rouxii* and *Rhodotorula mucilaginosa*, heat preadaptation could improve resistance to salt stress (Cheng et al., 2016; Wang et al., 2021). However, the mechanisms by which cross-protection occurs remains obscure. Previous studies have shown that two homologous transcription factors Msn2p and Msn4p, which are downstream of the HOG-MAPK cascade (Verghese et al., 2012), were associated with the basis of cross-stress resistance (Świȩciło, 2016). These findings reveal the important role of HOG-MAPK in cross-protection. In our previous research, we found that *StHOG1* is not only involved in osmotic stress but also related to pathogenicity (Li et al., 2016), indicating that there must be some relationship between osmotic stress reaction and pathogenicity in *S. turcica*. In accordance with our hypothesis, in the present study, we found the osmotic stress strengthens the formation of infection structures and enhances the pathogenicity of *S. turcica*. In addition, in the life cycle of phytopathogenic fungi, at conidium germination and the penetration phase, fungi are in a state of starvation, which can be regarded as starvation stress (Divon and Fluhr, 2007). During these periods, nutrients are entirely obtained from internal stores, of which glycerol is a major component (Thines et al., 2000; Weber et al., 2001). As for *S. turcica*, we speculate that exposure to osmotic stress strengthens its ability to synthesis glycerol via the HOG-MAPK pathway in *S. turcica*. Via pretreatment with hypertonic stress, fungi can quickly synthesize glycerol to meet the nutrient demand when they infect maize leaves. Subsequently, the glycerol produces the high turgor pressure required for appressorium, thereby increasing pathogenicity. Therefore, our experiment confirmed from a new perspective that osmotic stress as an important abiotic stress factor effects the growth and development, and pathogenicity by activating gene expression in HOG MAPK pathway, and the cell memory of osmotic stress can enhance the pathogenicity of *S. turcica*.

## Conclusion

In this study, qRT-PCR analysis indicated that the activation of the HOG-MAPK pathway and the expression of its downstream genes are quickly up-regulated at the transcriptional level to respond to osmotic stress. Importantly, it was demonstrated that osmotic stress affects the morphology of hypha, shortens the germination time of conidia, alters the structure of invasive hyphae, and enhances the pathogenicity of *S. turcica*. Genetic analysis revealed that *StFPS1*, a key gene downstream of HOG-MAPK pathway, influences the development of appressorium and the penetration ability to maize, but is not related to the virulence in *S. turcica*. In summary, our findings confirmed that there is a close relationship between osmotic stress response and pathogenicity in *S. turcica*.

## Data Availability Statement

The original contributions presented in the study are included in the article/Supplementary Material; further inquiries can be directed to the corresponding author/s.

## Author Contributions

SG and JD conceived and designed the experiments. YL participated in the experiments and wrote the manuscript. XG, ML, HS, QZ, XL, YF, and XZ performed the experiments. JH collect the experimental materials. All authors read and approved the final manuscript.

## Conflict of Interest

The authors declare that the research was conducted in the absence of any commercial or financial relationships that could be construed as a potential conflict of interest.

## Publisher’s Note

All claims expressed in this article are solely those of the authors and do not necessarily represent those of their affiliated organizations, or those of the publisher, the editors and the reviewers. Any product that may be evaluated in this article, or claim that may be made by its manufacturer, is not guaranteed or endorsed by the publisher.

## Footnotes

1 https://genome.jgi.doe.gov/Settu3/Settu3.home.html

## Supplementary Material

Supplementary Figure 1 Morphology of conidia culture under the three osmotic conditions (0.4 M NaCl, 0.4 M KCl, and 0.6 M sorbitol) and control (PDA) conditions.

Supplementary Figure 2 Observation of appressorium development of *S. turcica* grown for 3 h in 1.1 M NaCl, 1.1 M KCl, and 1.3 M sorbitol media. ap: appressorium; h: invading hyphae.

Supplementary Figure 3 The phenotype of *StFPS1* knockout mutants.

Supplementary Table 1 The primers used in this study.
