# Supplementary figures and images for: Effect of Osmotic Stress on the Growth, Development and Pathogenicity of Setosphaeria turcica
Source: Front Microbiol. 2021 Jul 23;12:706349. doi: 10.3389/fmicb.2021.706349 (PMC8342955; doi:10.3389/fmicb.2021.706349)

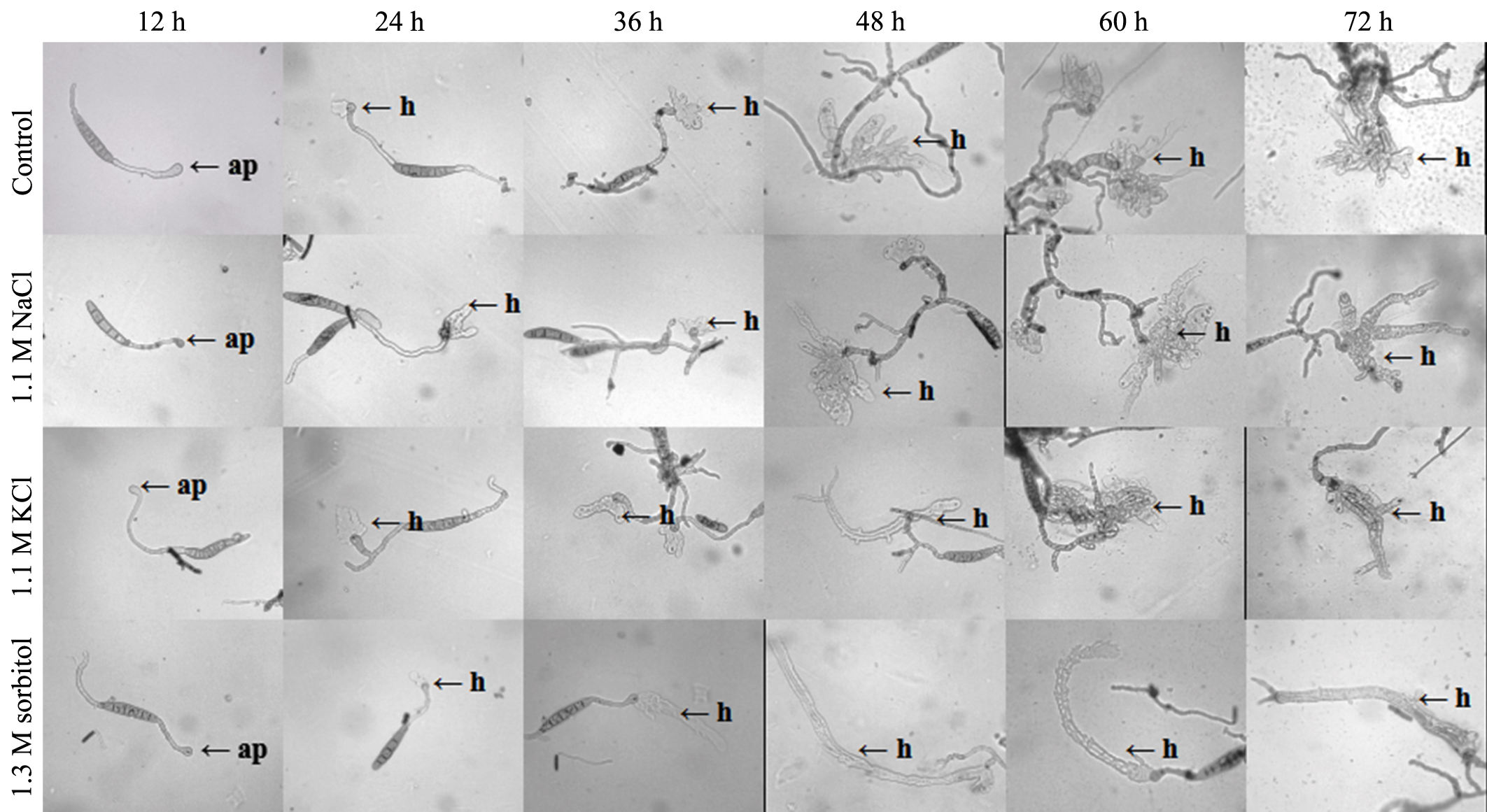

Supplement: Supplementary Figure 1 — Morphology of conidia culture under the three osmotic conditions (0.4 M NaCl, 0.4 M KCl, and 0.6 M sorbitol) and control (PDA) conditions. [file Image_1.TIF]

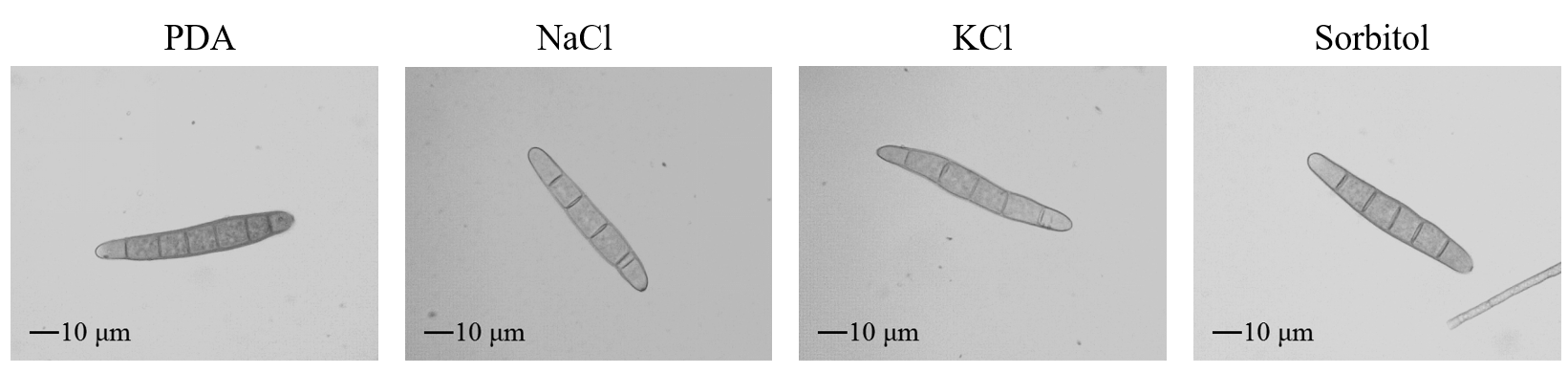

Supplement: Supplementary Figure 2 — Observation of appressorium development of S. turcica grown for 3 h in 1.1 M NaCl, 1.1 M KCl, and 1.3 M sorbitol media. ap: appressorium; h: invading hyphae. [file Image_2.TIF]

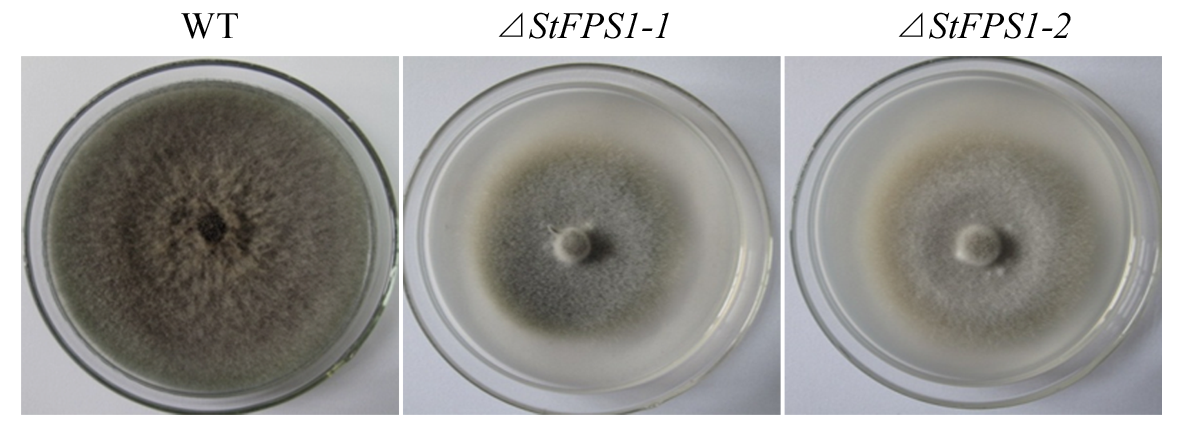

Supplement: Supplementary Figure 3 — The phenotype of StFPS1 knockout mutants. [file Image_3.TIF]
